# Open Mental Health: A Mental Health Eco System Developed in Collaboration With Communities

**DOI:** 10.1192/bjo.2022.348

**Published:** 2022-06-20

**Authors:** Jane Yeandle, Sarah Oke, Andreas Papadopoulos

**Affiliations:** Somerset NHS Foundation Trust, Somerset, United Kingdom

## Abstract

**Aims:**

Somerset drew upon the Community Mental Health (MH) Service Framework, and local experience and recognised that too often service user experience of mental health was beset with artificial barriers, gateways and eligibility with people sometimes needing to deteriorate before they got the vital support they needed. In addition, we recognised that the gap between secondary care and primary care was experienced by service users as a “cliff edge”. The radical redesign of mental offers in Somerset aimed to deliver an experience of “no wrong door” and where, via a partnership between health, social care and Voluntary, Community and Social Enterprise (VCSE), people's needs could be met; both in terms of specific mental health offers as well as tackling the wider determinants of mental ill health.

**Methods:**

OMH was launched early 2019, just before the pandemic, and so traditional project methodology did not always apply. Instead all partners focused on standing up the offer at pace in order to support the population within Somerset with the emotional and psychological consequences of the pandemic, and any pre existing challenges. The model was co-produced with Experts by Experience significantly contributing to the shape of the offers.

The model developed comprised of two key facets:

- A mental health offer, where the needs of individuals, families and carers are met by a range of health, social care and VCSE partners (including the wider determinants of mental well being such as finance / housing)
- A mental health eco system developed in collaboration with communities (including underserved communities), where support is available throughout geographical communities and communities of identity.

**Results:**

The project has delivered both quantitively and qualitatively. Over 4100 additional appointments are offered to people in Somerset per month and reported outcomes and evaluation has been very positive. Work is underway to collate further Patient Related Outcome Measures. Following a Realist Evaluation methodology, the researcher in residence has highlighted the culture changes that are key to delivering the aspirations of the Community MH Service framework. These include; the narrative of “no wrong door”, an increased and open range of offers and interventions, blended staffing models across traditional organisational boundaries, partnership working, the role of lived experience and the ambition of addressing inequalities.

**Conclusion:**

OMH, the product of Community MH Transformation in Somerset, is a radical and co produced redesign of MH services in partnership with the VCSE and Local Authority that has improved access and support to all people in Somerset.

